# Immune Dysregulation in Monogenic Inborn Errors of Immunity in Oman: Over A Decade of Experience From a Single Tertiary Center

**DOI:** 10.3389/fimmu.2022.849694

**Published:** 2022-04-06

**Authors:** Tariq Al Farsi, Khwater Ahmed, Jalila Alshekaili, Mahmood Al Kindi, Matthew Cook, Aliya Al-Hosni, Zainab Ansari, Iman Nasr, Nashat Al Sukaiti

**Affiliations:** ^1^Department of Pediatric Allergy and Clinical Immunology, The Royal Hospital, Muscat, Oman; ^2^Department of Microbiology and Immunology, Sultan Qaboos University Hospital, Muscat, Oman; ^3^Department of Immunology and Infectious Disease, John Curtin School of Medical Research, Australian National University, Canberra, NSW, Australia; ^4^Translational Research Unit, Department of Immunology, The Canberra Hospital, Canberra, NSW, Australia; ^5^Centre for Personalized Immunology (National Health and Medical Research Council (NHMRC) Centre of Research Excellence), John Curtin School of Medical Research, Australian National University, Canberra, NSW, Australia; ^6^Molecular Genetics, National Genetics Center, Muscat, Oman; ^7^Department of Adult Allergy and Clinical Immunology, The Royal Hospital, Muscat, Oman

**Keywords:** inborn errors of immunity, immunodeficiency, immune dysregulation, phenotype, genotype, children, adults, Omani

## Abstract

**Background:**

Inborn errors of immunity (IEIs) are being recognized as an important cause of morbidity and mortality in communities with a high frequency of consanguinity, such as Oman, and thus recessively inherited conditions. Various monogenic causes of IEI have been recently discovered; however, the disease phenotype may be variable and does not always include infection at presentation, leading to a delay in diagnosis and a poor outcome. It is now well recognized that immune dysregulation manifestations are observed in a significant proportion of patients with IEI and occasionally precede infection.

**Methods:**

Here, we retrospectively report the epidemiological, clinical, immunological, and molecular findings and outcomes from 239 patients with IEI who were diagnosed and managed at the Royal Hospital, Oman, from January 2010 to October 2021.

**Results:**

The estimated annual cumulative mean incidence of IEI was 25.5 per 100,000 Omani live births with a total prevalence of 15.5 per 100,000 Omani population. Both the high incidence and prevalence are attributed to the high rate of consanguinity (78.2%). Defects affecting cellular and humoral immunity including severe combined immunodeficiency (SCID), combined immunodeficiency (CID), and CID with syndromic features were the predominant defects in IEI (36%). Immune dysregulation was a prominent manifestation and occurred in approximately a third of all patients with IEI (32%), with a mean age of onset of 81 months and a mean diagnostic delay of 50.8 months. The largest percentage of patients who showed such clinical signs were in the category of diseases of immune dysregulation (41%), followed by predominantly antibody deficiency (18%). The overall mortality rate in our cohort was 25.1%, with higher death rates seen in CID including SCID and diseases of immune dysregulation.

**Conclusion:**

Immune dysregulation is a frequent manifestation of Omani patients with IEI. Early detection through raising awareness of signs of IEI including those of immune dysregulation and implementation of newborn screening programs will result in early intervention and improved overall outcome.

## Introduction

Inborn errors of immunity (IEIs) are a heterogeneous group of more than 400 primary immunodeficiency (PID) disorders, which are classified into 10 groups based on the involved pathophysiology, clinical phenotype, and genotype (1, 2). Prevalence studies based on the given clinical diagnosis have been conducted worldwide. Using disease registries, the prevalence is estimated to range from 1:8,500 to 1:100,000 people (3), according to the ethnic group and method used.

In a historical cohort study performed over a 31-year period using a public administrative healthcare database in the USA, the incidence rate of IEI was approximately 10.3/100,000 person-years, with predominantly antibody deficiency (PAD) accounting for 78% of all cases compared to 10.5% for combined immunodeficiency (CID) including severe combined immunodeficiency (SCID) (4). However, Asian registries especially those of Arabian gulf countries reported an IEI prevalence (per 100,000 children) of 20.2 in Kuwait (5), 4.7 in Qatar (6), and 7.2 in Saudi Arabia (7), with a predominance of CIDs affecting cellular and humoral immunity. In Oman, a previous report on PID among a pediatric cohort described a prevalence of 7/100,000 with an estimated incidence of 5.0/100,000. The main encountered IEIs were phagocytic defects (35%), followed by PAD (20.7%) and CID (17.8%) (8). In contrast, a study from our center demonstrated a higher incidence of SCID among an Omani pediatric population (4/100,000) compared to the earlier published data (9). Such epidemiological discrepancies in Oman can suggest underreporting of IEI in such retrospective reports.

A previous report from Oman described recurrent and severe infections as the only main clinical presentation of IEI (8). Infection is the cornerstone of the 10 warning signs of PID first proposed by the Jeffrey Modell Foundation in 1993 as a screening tool. However, with the expansion of knowledge on IEI, it is gradually becoming clearer that immune dysregulation is a major manifestation of this disease group. Indeed, later studies have demonstrated inadequate sensitivity of the initial 10 warning signs especially in infant and pediatric populations and have discussed the need for adjustment (10–12). In line with this, Thalhammer et al. (13) recommended adding immune dysregulation and syndromic features to the list of IEI warning signs to avoid diagnostic delay, which is associated with poorer health outcomes.

This study aimed to collect information on the frequency and manifestation of non-infectious phenotypes in the current extended cohort of pediatric and adult patients with IEI in Oman. Additionally, we provide more recent data on the incidence, prevalence, molecular diagnosis, and outcome of IEI in the Omani population.

## Methods

Data were extracted from electronic medical records by International Statistical Classification of Diseases (ICD)-10 codes (D80–D89) and were filled into Google forms and then an Excel sheet. A manual selection of patients was also performed, as some patients were not linked to ICD-10 codes. Further analysis was performed *via* Excel and GraphPad Prism-9 (GraphPad Software, Inc., San Diego, CA, USA). The results are expressed as means and medians with ranges and frequency (%) for continuous and categorical variables, respectively. The study was approved by the Research and Ethics Review and Approval Committee in the Ministry of Health, Sultanate of Oman.

### Patients

We performed an analysis of a cohort of 239 children and adults with IEI, who were seen and managed at the Royal Hospital—the main governmental tertiary hospital in Oman—from January 2010 to October 2021. The epidemiological details included the annual incidence per Omani live birth and the prevalence among the Omani population. The cumulative incidence was calculated by defining the mean of the yearly new diagnoses of IEI from the annual population of Omani live births obtained from local registries for the last 11 years. Given that most of our cohort were from the pediatric age group, the Omani live birth group was considered a reasonable population at risk to extrapolate a relatively true incidence. However, prevalence included all patients with IEI who were diagnosed from the Omani population over the last 11 years. Additionally, other information such as demographics (sex and age), family history, and clinical manifestations of infection, immune dysregulation, and malignancy, as well as molecular diagnosis, long-term events, and outcomes were collected into an electronic database. The adopted diagnostic criteria were based on The European Society for Immunodeficiencies (ESID) Registry Working Definitions for the Clinical Diagnosis of IEI (14). The classification and subclassification of IEI were based on the Human Inborn Errors of Immunity: 2019 Update of the International Union of Immunological Societies (IUIS) Phenotypical Classification (1, 2).

### Age Categories and International Union of Immunological Societies (IUIS) Classification

The age of onset and age of diagnosis were grouped into the following eight categories: 0–2, 3–5, 6–10, 11–15, 16–20, 21–30, 31–40, and >40 years. The phenotypic and genotypic classification was based on the main categories of IEI described in the IUIS (1, 2). Autoinflammatory disorders and syndromes of familial hemophagocytic lymphohistocytosis were excluded from the analysis due to the unavailability of patient’s details. Unclassified IEI was defined when the clinical phenotype and genotype did not fit the above classes. Diseases with possible contributions to IEI and those that were yet to be further studied were highlighted.

### Clinical Presentation, Disease Manifestation, and IEI Complications

Clinical presentations were divided into infection, immune dysregulation, malignancy, or asymptomatic. Detailed infectious and non-infectious disease manifestations and complications are listed. We performed a focused analysis on immunodysregulatory disorders to reveal specific characteristics with IEI classification, phenotype, genotype, diagnostic delay, and outcome.

### Laboratory Evaluation

The laboratory evaluation included full blood count (hemoglobin level and total lymphocyte, neutrophil, eosinophil, and platelet count), basic lymphocyte subsets [T, B, and Natural killer (NK) cell count] and total serum immunoglobulin levels (IgG, IgA, and IgM, IgE). Moreover, we also utilized a specific antibody panel for tetanus, diphtheria toxin, *Haemophilus influenzae* type B, pneumococcal antibodies, hepatitis B, measles, mumps, rubella and varicella, and dihydrorhodamine (DHR) assay for neutrophil function, a functional complement assay for classical and alternative hemolytic pathways and a CD11/CD18 expression assay for adhesion defect when clinically indicated.

### Genotype

Molecular diagnosis was obtained through whole-exome sequencing, Sanger sequencing, targeted mutation analysis, fluorescent *in situ* hybridization (FISH), or microarray. Confirmed genetic diagnosis was determined when patient had a positive pathogenic or likely pathogenic genetic change that could explain the clinical phenotype. A note was made on the proportion of positive mutations according to the results of a commercial lab, National Genetic Centre (NGC), in Oman or *via* the help of external expert research laboratories. The novelty of the mutation detected was confirmed by reviewing the query interface on the ClinVar database. Genetic workup for patients with Predominantly Antibody Deficiency (PAD) (IUIS class III) was not frequently requested due to limited resources.

### Long-Term Events and Outcome

The patients’ clinical outcomes were determined after reviewing the medical record system, noting long-term infectious and non-infectious manifestations. We also reported on the treatment options received, and information on the recent status was defined as follows: alive and well, alive with complications, need for Intensive Care Unit (ICU) admission, defaulted patients, mortality, and the contributions to mortality.

## Results

The Sultanate of Oman is the second largest territory in the Arabian Peninsula with an area of 120,000 square miles, a total coastal border of 3,165 km, and a total population of 4,471,148 in 2020. The geographical location has led to open access to immigration and trading from various ancient civilizations in Asia and North Africa. Oman has two major tertiary referral centers for IEI, and one bone marrow transplant unit, which is limited to four beds. The Bacillus Calmette–Guerin (BCG) vaccine is a routine immunization at birth for all newborns. There is currently no newborn screening program for T- and B-cell deficiencies, and referrals from primary physician to tertiary units are based on clinical suspicion and previous family history of IEI. Most patients were referred from government primary or secondary hospitals (68.6%). This is the first report to describe our IEI cohort over the last 11 years.

### Patient Demographics

Two hundred thirty-nine (n = 239) patients were diagnosed and managed at the Royal Hospital over a period of 11 years (January 2010–October 2021). The estimated annual cumulative mean incidence of IEI was 25.5 (range: 20.0–29.8) per 100,000 Omani live births, and combined with the Middle East and North Africa region (MENA) Omani cohort (15), the total prevalence of IEI was 15.5/100,000 Omani population (**Table 1**). There were slightly more boys in the registry (n = 139, 58.1%, and M/F: 1.4), and 194 (81.2%) patients were under 18 years of age. More than two-thirds of the cohort (n = 187, 78.2%) were born to consanguineous parents. Among the relatives of patients with IEI, a positive family history of recurrent infections was detected in 100 patients (41.8%) compared to a positive family history immune dysregulation (n = 38, 15.9%) and a positive family history of malignancy (n = 17, 7.1%). Regardless of the age of disease onset, there was a diagnostic delay. The mean and median age differences between the onset of symptoms and diagnosis were 41.7 and 15 months (range: 1–453), respectively. Details of the age of onset and diagnosis for Omani patients with IEI are shown in **Table 2**.

**Table 1:** Combined data showing the prevalence of IEI in Omani patients according to IUIS classification.

Type (IUIS)	MENA Omani cohort 2021	Current cohort	Total
Phagocytic defects (V)	59	35	94
SCID/CID (I)	38	55	93
PAD (III)	33	40	73
Syndromic CID (II)	35	31	66
Innate immune defects (VI)	5	35	40
Immune dysregulation (IV)	8	32	40
Complement deficiencies (VIII)	7	6	13
Phenocopies (X)	NR	1	1
Unclassified to IUIS	NR	4	4
Total number	185	239	424
Prevalence/100,000	6.8	8.7	15.5

IEI, Inborn Errors of Immunity; IUIS, International Union of Immunological Societies; MENA, Middle East and North Africa region; SCID, Severe Combined Immunodeficiency; CID, Combined Immunodeficiency; PAD, Predominantly Antibody Deficiency; NR, Not Reported.

**Table 2:** Distribution of patients with IEI in the categories of age according to onset and diagnosis.

Age in years	Diagnosis								Total	%
Onset	0–2	3–5	6–10	11–15	16–20	21–30	31–40	>40		
0–2	136	18	23	7	2		1		187	78.2
3–5		5	9	2		2			18	7.8
6–10			2	2	2	1			7	3.0
11–15				2	4	1	1		8	3.4
16–20					2	4	1		7	3.0
21–30						5	1		6	2.6
31–40							5	1	6	2.6
Total	136	23	34	13	10	13	9	1	239	
%	56.9	9.6	14.2	5.6	4.3	5.6	3.9	0.4		100

IEI, Inborn Errors of Immunity.

### Clinical Phenotype

Most patients presented with infection (n = 219, 91.6%) at clinical presentation, among whom 40 of them (16.7%) also had symptoms of immune dysregulation. Five patients presented with malignancy (2%), and the remainder were asymptomatic (n = 5, 2%) at clinical presentation as they were familial.

### Infection

**Figure 1:**
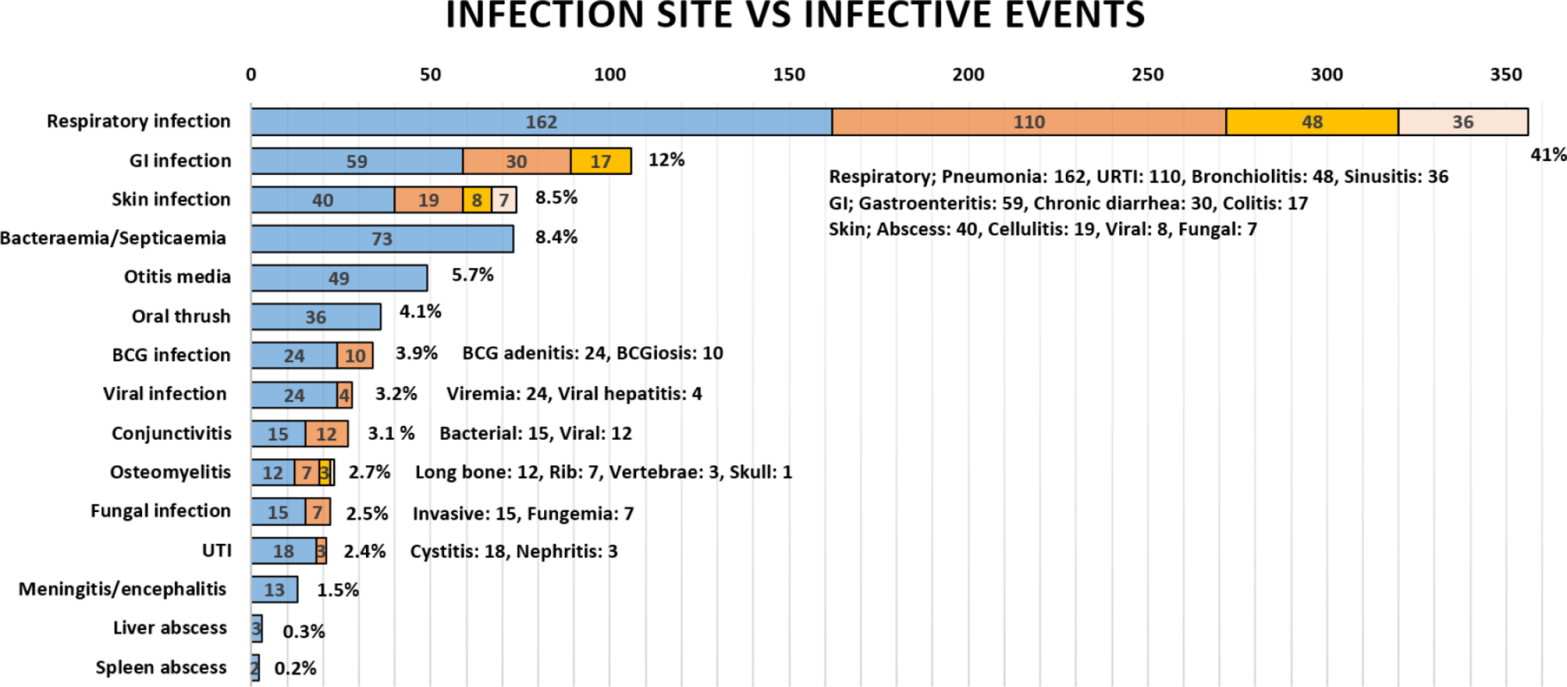
The frequency of infections in Omani patients with IEI according to infection site. GI, gastrointestinal; BCG, Bacillus Calmette–Guerin; UTI, urinary tract infection; URTI, upper respiratory tract infection.

The mean age of infection episode onset was 42.6 months (range: 1–480). The documented infectious etiology varied but mainly included Gram-negative (n = 143, 61.6%) and Gram-positive (n = 130, 56%) bacterial infections; this was followed by viral infections including the common viruses (n = 114, 49.1%), cytomegalovirus (n = 29, 12.5%), Epstein–Barr virus, (n = 16, 6.9%), and adenovirus (n = 13, 5.6%); fungal infections, including non-filamentous (n = 37, 15.9%), filamentous (n = 29, 12.5%), and dimorphic fungi (n = 13, 5.6%); and mycobacterial infections with *Mycobacterium bovis*, including BCG strain (n = 41, 17.7%), *Mycobacterium tuberculosis* (n = 3, 1.3%), and atypical mycobacterium (n = 3, 1.3%). Of note, most patients (n = 231, 96.7%) received BCG vaccination at birth before diagnosis. The lowest reported infection etiology was protozoal agents (n = 5, 2.2%). In regard to the frequency of infectious etiology among patients with infections (n = 219), approximately half (n = 106, 48.4%) had up to two etiologies, a third (n = 64, 29.2%) had three etiologies, and a fifth have four or more etiologies (n = 49, 22.3%). The most commonly reported infection site was the respiratory tract (41.1%), followed by gastrointestinal (12.2%), cutaneous (8.5%), bacteremia and septicemia (8.4%), and other infections. The frequency of clinical presentations is grouped under infections, and the involved sites are illustrated in **Figure 1**.

### Immunodysregulation

Immunodysregulatory manifestations were prominent in 77 patients (32.2%), among whom 27 patients (35%) died and 5 patients received hematopoietic stem cell transplantation (HSCT), as it was clinically indicated for their phenotype and genotype (DCLRE1C, RAB27A, LYST, LRBA, and XIAP). The mean and median age of onset of immune dysregulation was 81 and 25 (range: 1–480) months, respectively. The mean and median diagnostic delay in patients with immunodysregulatory manifestations was 50.8 and 19 months, respectively. Thirty-two patients were categorized as IEI with immunodysregulation (13.4%) according to IUIS classification, among whom the genotype of 21 patients (8.8%) was identified. The recorded immunodysregulatory manifestations included Omenn’s syndrome, recurrent oral ulcers, autoimmune cytopenia, lymphoproliferation, autoimmune enteropathy, autoimmune thyroid disease, autoimmune nephropathy, diabetes mellitus type 1, autoimmune arthritis, systemic lupus erythematosus, hemophagocytic lymphohistiocytosis, interstitial lung disease, autoimmune eye disease, and others. The features of the genotype, phenotype, and outcomes of patients with immune dysregulation are detailed in **Table 3**. Patients with non-infective lymphoproliferation were found to have splenomegaly (n = 32, 13.4%), hepatomegaly (n = 29, 12.1%), generalized (n = 25, 10.5%), and localized lymphadenopathy (n = 13, 5.4%).

**Table 3:** Summary describes the immunodysregulatory manifestations of patients with IEI according to IUIS category, genotype, phenotype, and outcome.

Classification	Gene	Immunodysregulation type	Median (months)	Outcome
*Immunodeficiencies affecting cellular and humoral immunity (Ia)*	JAK3 (2)	Omenn’s syndrome	Immunodysregulation onset: 8 Diagnostic delay: 3.5	Died (2)
	RAG1 (2)	Omenn’s syndrome	Immunodysregulation onset: 0.25 Diagnostic delay: 1.5	Died (2)
	CD3E (1)	Omenn’s syndrome	Immunodysregulation onset: 2.5 Diagnostic delay: 0.5	Died (1)
	DCLRE1C (1)	Omenn’s syndrome	Immunodysregulation onset: 2 Diagnostic delay: 1	Alive (1) HSCT (1)
	NA (1)	Omenn’s syndrome	Immunodysregulation onset: 6 Diagnostic delay: 4	Died (1)
*Immunodeficiencies affecting cellular and humoral immunity (Ib)*	STK4 (2)	Lymphoproliferation. Autoimmune eye disease. Atopic dermatitis.	Immunodysregulation onset: 78 Diagnostic delay: 99	Alive (2)
	CD40LG (1)	Recurrent oral ulcers. Autoimmune enteropathy.	Immunodysregulation onset: 16 Diagnostic delay: 1	Alive (1)
*Combined immunodeficiencies (II)*	TTC7A (2)	Autoimmune enteropathy	Immunodysregulation onset: 1 Diagnostic delay: 3	Died (2)
	CHD7 (2)	Autoimmune thyroid disease. Autoimmune nephropathy. CNS vasculitis.	Immunodysregulation onset: 73 Diagnostic delay: 25.5	Died (2)
	SPINK5 (3)	Lymphoproliferation. Autoimmune nephropathy. Ichthyosis	Immunodysregulation onset: 1 Diagnostic delay: 4	Alive (1) Died (2)
	NA (4)	Lymphoproliferation. Autoimmune cytopenia. Autoimmune thyroid disease. Hemophagocytic lymphohistocytosis. Autoimmune enteropathy. Atopic dermatitis. Vitiligo	Immunodysregulation onset: 25 Diagnostic delay: 13.5	Alive (3) Died (1)
*Predominantly antibody deficiencies (III)*	NA (14)	Recurrent oral ulcers. Autoimmune cytopenia. Lymphoproliferation. Autoimmune enteropathy. Autoimmune nephropathy. Autoimmune eye disease. Autoimmune thyroid disease. Diabetes mellitus type 1. Autoimmune encephalitis. Autoimmune arthritis. Systemic lupus erythematosus. Serositis.	Immunodysregulation onset: 178 Diagnostic delay: 48	Alive (12) Died (2)
*Diseases of immune dysregulation (IVa)*	AP3D1 (4)	Lymphoproliferation	Immunodysregulation onset: 2 Diagnostic delay: 35	Died (3) Alive (1)
	RAB27A (3)	Lymphoproliferation. Autoimmune enteropathy. Autoimmune cytopenia. Hemophagocytic lymphohistocytosis.	Immunodysregulation onset: 36 Diagnostic delay: 60	Alive (1) HSCT (1) Died (2)
	AP3B1 (3)	Lymphoproliferation. Autoimmune eye disease.	Immunodysregulation onset: 14 Diagnostic delay: 9.5	Alive (2) Died (1)
	PRKCD (1)	Nil so far	Diagnostic delay: 22	Alive (1)
	LYST (1)	Lymphoproliferation. Hemophagocytic lymphohistocytosis.	Immunodysregulation onset: 20 Diagnostic delay: 3	HSCT (1) Died (1)
*Diseases of immune dysregulation (IVb)*	LRBA (5)	Lymphoproliferation. Autoimmune cytopenia. Autoimmune thyroid disease. Diabetes mellitus type 1. Interstitial lung disease. Autoimmune eye disease. Alopecia areata. Atopic dermatitis.	Immunodysregulation onset: 2 Diagnostic delay: 7	Alive (5) HSCT (1)
	XIAP (2)	Lymphoproliferation. Hemophagocytic lymphohistocytosis.	Immunodysregulation onset: 120 Diagnostic delay: 12	Alive (2) HSCT (1)
	IL2RA (1)	Lymphoproliferation. Autoimmune cytopenia. Autoimmune thyroid disease. Diabetes mellitus type 1. Autoimmune hepatitis. Interstitial lung disease.	Immunodysregulation onset: 1 Diagnostic delay: 10	Died (1)
	FOXP3 (1)	Recurrent oral ulcers. Lymphoproliferation. Systemic lupus erythematosus.	Immunodysregulation onset: 180 Diagnostic delay: 96	Alive (1)
*Diseases of immune dysregulation (IV)*	NA (11)	Recurrent oral ulcers. Autoimmune thyroid disease. Autoimmune cytopenia. Hemophagocytic lymphohistocytosis. Lymphoproliferation. Autoimmune hepatitis. Autoimmune nephropathy. Diabetes mellitus type 1. Autoimmune enteropathy. Autoimmune joint disease. Systemic lupus erythematosus. Alopecia areata. Atopic dermatitis.	Immunodysregulation onset: 17 Diagnostic delay: 6	Alive (9) Died (2)
*Congenital functional defects of phagocyte (V)*	NCF1 (2)	Recurrent oral ulcers. Lymphoproliferation. Autoimmune enteropathy. Autoimmune eye disease.	Immunodysregulation onset: 16.5 Diagnostic delay: 48.5	Alive (2)
*Defects in intrinsic and innate immunity (VI)*	STAT1-GOF (2)	Autoimmune thyroid disease	Immunodysregulation onset: 66 Diagnostic delay: 261	Alive (2)
	NA (1)	Autoimmune arthritis	Immunodysregulation onset: 468 Diagnostic delay: 12	Alive (1)
*Complement deficiencies (VIII)*	NA (1)	Lymphoproliferation. Autoimmune cytopenia. Hemophagocytic. Lymphohistocytosis.	Immunodysregulation onset: 144 Diagnostic delay: 28	Alive (1)
*Phenocopies of PID (X)*	NA (1), [Good syndrome]	Autoimmune cytopenia.	Immunodysregulation onset: 396 Diagnostic delay: 120	Died (1)
*Unclassified IEI*	ZNFX1 (1)	Recurrent oral ulcers. Autoimmune cytopenia. Hemophagocytic. Lymphohistocytosis.	Immunodysregulation onset: 3 Diagnostic delay: 9	Died (1)
*Potential IEI*	LAT2 (1)	Autoimmune thyroid disease. Autoimmune nephropathy. Autoimmune eye disease. Autoimmune arthritis. Atopic dermatitis. Ichthyosis.	Immunodysregulation onset: 48 Diagnostic delay: 396	Alive (1)
	EBF3 (1)	Autoimmune enteropathy. Atopic dermatitis.	Immunodysregulation onset: 96 Diagnostic delay: 164	Alive (1)

IEI, Inborn Errors of Immunity; IUIS: International Union of Immunological Societies; JAK3, Janus Tyrosine Kinase 3; RAG1, Recombination Activating Gene 1; CD3E, Cluster of Differentiation 3 Epsilon; DCLRE1C, DNA Cross-Link Repair 1C; NA, Not Available; STK4, Serine/Threonine Kinase 4; CD40LG, Cluster of Differentiation 40 Ligand; TTC7A, Tetratricopeptide Repeat Domain 7A; CHD7, Chromodomain Helicase DNA-binding protein 7; SPINK5, Serine Peptidase Inhibitor Kazal Type 5; AP3D1, Adaptor Related Protein Complex 3 Delta 1; RAB27A, Ras-related protein Rab 27A; AP3B1, Adaptor-related Protein complex 3 Beta 1; PRKCD, Protein Kinase C Delta; LYST, Lysosomal Trafficking regulator; LRBA, Lipopolysaccharide-Responsive and Beige-like Anchor; XIAP, X-linked Inhibitor of Apoptosis; IL2RA, Interleukin-2 Receptor subunit Alpha; FOXP3, Forkhead Box P3; NCF1, Neutrophil Cytosolic Factor 1; STAT1, Signal Transducer and Activator of Transcription 1; GOF, Gain Of Function; ZNFX1, Zinc Finger NFX1-type containing 1; LAT2, L-type Amino acid Transporter 2; EBF3, Early B-cell Factor 3. Unclassified IEI: a genetic variant that cause a novel IEI that is not yet classified into the current categories of IUIS. Potential IEI: a genetic variant with possible causality to IEI, and yet to be studied and confirmed on Humans.

### Malignancy

The mean and median age of malignancy episode onset was 54 and 36 months, respectively (range: 12–114). A malignant process was reported as Hodgkin’s lymphoma (n = 3, 1.3%), non-Hodgkin’s lymphoma (n = 3, 1.3%), and other malignancies (n = 2, 0.8%).

### Immunological Evaluation

In more than a third of the patients, the basic immune workup revealed T-cell lymphopenia (n = 88, 37.9%) and B-cell lymphopenia (n = 70, 30.1%). Other observed cell line abnormalities excluding autoimmune cytopenia included anemia (n = 70, 30.1%), neutropenia (n = 45, 19.4%), thrombocytopenia (n = 34, 14.7%), and eosinophilia (n = 5, 2.2%). Various serum immunoglobulin abnormalities were detected including hypogammaglobulinemia (n = 55, 23.7%), hypergammaglobulinemia (n = 30, 12.9%), hyper-IgE (n = 28, 12.1%), dysgammaglobulinemia (n = 26, 11.2%), agammaglobulinemia (n = 18, 7.8%), specific antibody deficiency (n = 7, 3%), IgG subclass deficiency (n = 6, 2.6%), IgA deficiency (n = 2, 0.9%), and hyper-IgM (n = 2, 0.9%). Other reported immunological findings were abnormal DHR123 assay (n = 33, 14.2%), abnormal complement number and function assay (n = 6, 2.6%), and abnormal expression of CD11/CD18 on flow cytometry (n = 1, 0.4%).

### Genotype

Genetic diagnosis was confirmed in 120 patients (50%), with a mean and median age of 59 and 21 months, respectively (range: 0.5–492). However, the median age (in months) of a delay in obtaining a genotype was 69 for class VI, 26.5 for class V, 21.5 for class III, 14 for class IV, 13.5 for class II, and 6 for class I. Homozygosity was paramount, as 90% of all genotypes were autosomal recessive in inheritance (n = 108). In addition to the known mutations associated with the described groups of IEI, we reported three mutations in genes described to cause IEI that are not yet classified into IUIS [LIFR (16), DIAPH1 (17), ZNFX1 (18)]. Additionally, there were two homozygous variants [LAT2 (19) and EBF3 (20)] in genes implicated in immune function in mice that require further immunological functional studies to support a disease phenotype–genotype correlation in humans (**Table 4**). The modalities used for confirming the molecular diagnosis were whole-exome sequencing and next-generation sequencing (n = 70), Sanger sequencing as targeted mutation analysis (n = 38), and FISH and microarray (n = 12). We identified the genotype using a commercial lab (n = 92, 38.5%), the NGC in Oman (n = 20, 8.4%), and through external expert research laboratories (n = 8, 3.3%). The percentage of familial cases among IUIS classes was 67.6% for class V, 61.8% for class I, 46% for class II, 33.3% for class IV, 16.7% for class VI, and 7.5% for class III. No genetic diagnosis was obtained after a patient’s death.

**Table 4:** Summary of the identified genotype of IEI cohort shown in categories of IUIS, unclassified IEI, and potentially disease-causing variants.

IUIS class	Gene	Mutation	Protein effect	Exon	Type	Zygosity	Novelty
***Ia***	IL7R	c.616c>T	p.(Arg206)	5	Nonsense	Homozygous	Novel, not reported
	ADA	c.815G>A	p.(Trp272)	9	Stop-gain	Homozygous	Novel, not reported
	ADA	c.910del	p.(Leu304Trpfs7)	10	Frameshift	Homozygous	Novel, not reported
	ADA	c.646G>A	p.(Gly216Arg)	7	Missense	Homozygous	VCV000001968, Pathogenic
	JAK3	c.1613G>A	p.(Gly538Asp)	12	Missense	Homozygous	Novel, not reported
	JAK3	c.2490+1G>A, c.1645C>T	p.(Arg549)	18	Nonsense	Compound Heterozygous	VCV001066836, likely pathogenic. VCV000803542, pathogenic
	RAG1	c.1187G>A	p.(Arg396His)	2	Missense	Homozygous	VCV000013146, Pathogenic
	RAG1	c.2924G>C	p.(Arg975Pro)	2	Missense	Homozygous	VCV000013146, pathogenic
	DCLRE1C	c. 404G>A	p.(Gly135Glu)	6	Missense	Homozygous	Novel, not reported
	CD3E	c.351A>C	p.(Arg117Ser)	6	Frameshift	Homozygous	Novel, not reported
	NHEJ1	c.530-1G>A	Intronic acceptor splice site	5	Substitution	Homozygous	Novel, not reported
	IL2RG	c.854G>A	p.(Arg285Gln)	6	missense	Hemizygous	VCV000010026, Pathogenic
***Ib***	DOCK8	c.53+240A>C	p.(Lys43Arg)	1	Missense	Homozygous	VCV000676901,
	CIITA	c.183_184delinsCT	p.(Lys62)	2	Missense	Homozygous	Novel, not reported
	CIITA	c.3212T>C	p.(Met1071Thr)	17	Missense	Homozygous	Novel, not reported
	CIITA	chr16:10907596-10907830del	234 bp Deletion	11	Frameshift	Homozygous	Novel, not reported
	RFX5	c.446G>A	p.(Arg149Gln)	7	Missense	Homozygous	VCV000007648, VUS
	RFXANK	c.634C>T	p.(Arg212Ter)	8	Nonsense	Homozygous	VCV001074711, Pathogenic
	MSN	c.796-3c>G	Intronic	8	Substitution	Hemizygous	Novel, not reported
	MAP3K14	c.1694 C>T	p.(Pro565Leu)	11	Missense	Homozygous	Novel, not reported
	CD40LG	c.430G>T	p.(Gly144)	5	Nonsense	Hemizygous	Novel, not reported
	CD40LG	c.506A>G	p.(Tyr169Cys)	5	Missense	Hemizygous	VCV000191254, likely pathogenic
	STK4	C.1A>T	p.Met1Leu	1	Splice	Homozygous	Novel, not reported
***IIa***	ATM	c.7481dup	p.(Asn2494Lysfs6)	50	Frameshift	Homozygous	Novel, not reported
	ATM	c.3277delA	p.(Ile1093Serfs16)	22	Frameshift	Homozygous	Novel, not reported
	CHD7	c.8416C>G	p.(Leu2806Val)	38	Missense	Homozygous	VCV000095814
***IIb***	STAT3	c.2132T>G	p.(Ile711Thr)	23	Missense	Heterozygous	VCV000803390, likley pathogenic
	STAT3	c.1110-2A>G	p.(Arg382Trp)	12	Substitution	Heterozygous	Novel, not reported
	STAT3	c.550+1C>T	Donor splice site	Intron 18	Splicing	Heterozygous	Novel, not reported
	SPINK5	c. 1887+1G>A	hg19:Chr5:147492498	Intron 20	Splicing	Homozygous	Novel, not reported
	SPINK5	c.2557C>T	p.(Arg853)	27	Stop-gain	Homozygous	VCV000623372, pathogenic
	TTC7A	c.122del	p.(Met41Serfs38)	1	Frameshift	Homozygous	Novel, not reported
	TTC37	c.2114+5G>A	Intronic	20	Splicing	Homozygous	Novel, not reported
***IIIa***	BTK	chrX:100601487-100614367		11_19	Deletion	Hemizygous	Novel, not reported
	BTK	c.828dup	p.(IIe277Tyrfs15)	9	Frameshift	Hemizygous	Novel, not reported
	CD79A	c.91C<T	p.(Gln31)	2	Nonsense	Homozygous	Novel, not reported
***IVa***	LYST	Chr1:235840380-235840919		49-50	Deletion	Homozygous	Novel, not reported
	RAB27A	c.340 A>G	p.(IIe114Val)	1_5	Missense	Homozygous	Novel, not reported
	RAB27A	c.153+2T>C	Intrionic	Intron 3	Splicing	Homozygous	Novel, not reported
	AP3B1	c.12_13delTA	p.(Asn4LysfsStop6)	1	Frameshift	Homozygous	Novel, not reported
	AP3B1	c.205-1G>C	Acceptor splice site	Intron 2	Splicing	Homozygous	Novel, not reported
	AP3D1	c.205-1G>C	Acceptor splice site	3	Splicing	Homozygous	Novel, not reported
	PRKCD	c.723del	p.(Thr242Profs14)	9	Frameshift	Homozygous	Novel, not reported
***IVb***	LRBA	c.3811C>T	p.(Arg1271)	23	Nonsense	Homozygous	VCV000574254, Pathogenic
	LRBA	chr4:151814193-151935794		1_17	Deletion	Homozygous	Novel, not reported
	IL2RA	c.418T>C	p.(Tyr140His)	4	Substitution	Homozygous	Novel, not reported
	FOXP3	c.1186G>C	P.(Val396Leu)	10	Missense		Novel, not reported
	XIAP	c.990_991del	p.(Leu331Argfs18)	4	Frameshift	Hemizygous	Novel, not reported
***Va***	SBDS	c.258+2T>C,	Donor splice site	Intron 2	Splicing,	Compound Heterozygous	VCV000003196, pathogenic
		c.183_184delinsCT	p.(Lys62)	Exon 2	Nonsense		VCV000003195, pathogenic
***Vb***	NCF1	c.579G>A	P.(Trp193)	7	Stop-gain	Homozygous	VCV00042699, Pathogenic
	CYBA	c.314T>G	p.(Leu105Arg)	165	Missense	Homozygous	Novel, not reported
***VIa***	MYD88	c.814C>T	p.(Arg272Ter)	5	Nonsense	Homozygous	Novel, not reported
	IFNGR2	c..175+102del	Intronic	1	Deletion	Homozygous	Novel, not reported
	STAT1	c.820C>T	p.(Arg274Trp) GOF	8	Missense	Heterozygous	VCV000030083, Pathogenic
	STAT1	c.800C>T	p.(Ala267Val) GOF	8	Missense	Heterozygous	VCV000030084, likely pathogenic
	IL17RC	c.1414C>T	p.(Arg472)	17	Nonsense	Homozygous	Novel, not reported
***VIb***	STAT1	c.778C>T	p.(Q260X)	8	Nonsense	Homozygous	Novel, not reported
	IL12RB1	Chr19:18186575: c.804_805insT	p.(Val269fs)	9	Frameshift	Homozygous	Novel, not reported
	TYK2	c.1969G>A	p.(E657K)	14	Missense	Homozygous	Novel, not reported
***Unclassified IEI***	LIFR	c.653dup	p.(Glu219fs)	6	Duplication frameshift	Homozygous	VCV000281444, Pathogenic
	DIAPH1	c.2769del	p.(Phe923Leufs4)	21	Frameshift	Homozygous	VCV000217753, Pathogenic
	ZNFX1	c.1405G>C	p.(Val469Leu)	1_12	Missense	Homozygous	Novel, not reported
***Potential IEI***	LAT2	c.95G>T	p.(Gly32Val)	3	Missense	Homozygous	Novel, not reported
	EBF3	c.509G>T	p.(Cys170Phe)	6	Missense	Heterozygous	Novel, not reported

Immunodeficiencies affecting cellular and humoral immunity (I). Combined immunodeficiencies (II). Predominantly antibody deficiencies (III). Diseases of immune dysregulation (IV). Congenital functional defects of phagocyte (V). Defects in intrinsic and innate immunity (VI).

IEI, Inborn Errors of Immunity; IUIS: International Union of Immunological Societies; IL7R, Interleukin 7 Receptor; ADA, Adenosine Deaminase; JAK3, Janus Tyrosine Kinase 3; RAG1, Recombination Activating Gene 1; DCLRE1C, DNA Cross-Link Repair 1C; CD3E, Cluster of Differentiation 3 Epsilon; NHEJ1, Non-Homologous End-Joining factor 1; IL2RG, Interleukin-2 Receptor subunit Gamma; DOCK8, Dedicator of Cytokinesis 8; CIITA, Class II major histocompatibility complex Transactivator; RFX5, Regulatory Factor X5; RFXANK, Regulatory Factor X-associated Ankyrin-containing; MSN, Moesin; MAP3K14, Mitogen-Activated Protein 3 Kinase 14; CD40LG, Cluster of Differentiation 40 Ligand; STK4, Serine/Threonine Kinase 4; ATM, Ataxia Telangiectasia Mutated; CHD7, Chromodomain Helicase DNA-binding protein 7; STAT3, Signal Transducer and Activator of Transcription 3; SPINK5, Serine Peptidase Inhibitor Kazal Type 5; TTC7A, Tetratricopeptide Repeat Domain 7A; TTC37, Tetratricopeptide repeat domain 37; BTK, Bruton's Tyrosine kinase; CD79A, Cluster of Differentiation 79A; LYST, Lysosomal Trafficking regulator; RAB27A, Ras-related protein Rab 27A; AP3B1, Adaptor-related Protein complex 3 Beta 1; AP3D1, Adaptor Related Protein Complex 3 Delta 1; PRKCD, Protein Kinase C Delta; LRBA, Lipopolysaccharide-Responsive and Beige-like Anchor; L2RA, Interleukin-2 Receptor subunit Alpha; FOXP3, Forkhead Box P3; XIAP, X-linked Inhibitor of Apoptosis; SBDS, Shwachman-Bodian-Diamond Syndrome; NCF1, Neutrophil Cytosolic Factor 1; CYBA, Cytochrome B-245 Alpha chain; MYD88, Myeloid Differentiation primary response 88; IFNGR2, Interferon Gamma Receptor 2; STAT1, Signal Transducer and Activator of Transcription 1; GOF, Gain Of Function; IL17RC, Interleukin-17 Receptor C; IL12RB1, Interleukin 12 Receptor subunit Beta 1; TYK2, Tyrosine-protein Kinase 2; LIFR, Leukemia Inhibitory Factor Receptor; DIAPH1, Diaphanous Homolog 1; ZNFX1, Zinc Finger NFX1-type containing 1; LAT2, L-type Amino acid Transporter 2; EBF3, Early B-Cell Factor 3; Arg, Arginine; Trp, Tryptophan; del, deletion; ins, insertion; dup, dublication; Leu, Leucine; Gly, Glycine; Lys, Lysine; Met, Methionine; Thr, Threonine; bp, base pair, Gln, Glutamine; Val, Valine; Pro, Proline; Tyr, Tyrosine; His, Histidine; Asn, Asparagine; Ile, Isoleucine; Ser, Serine; Val, Valine; Ter, Terminator, Ala, Alanine; Glu, Glutamic acid; Phe, Phenylalanine; Cys, Cysteine. Unclassified IEI: a genetic variant that cause a novel IEI that is not yet classified into the current categories of IUIS. Potential IEI: a genetic variant with possible causality to IEI, and yet to be studied and confirmed on Humans.

### Long-Term Events and Outcome

One hundred thirty-one (54.8%) patients failed to gain adequate weight during their early life. Chronic lung disease constituted a major complication of IEI. Bronchiectasis (n = 62, 25.9%), bronchial asthma (n = 27, 11.3%), and interstitial lung disease (ILD; n = 12, 5%) were the most commonly reported lung complications. End-organ damage (n = 54, 22.6%) was recorded for the brain (n = 14, 5.9%), hearing (n = 12, 5%), liver (n = 8, 3.3%), bone marrow (n = 6, 2.5%), kidney (n = 6, 2.5%), skin (n = 4, 1.5%), and eye (n = 4, 1.5%). In comparison to the overall median age of diagnostic delay (15 months), further delay was noted for patients with the following: bronchiectasis, 60 (2–453); combined end-organ damage, 32.5 (1–204); and bronchial asthma, 28.5 (1–396); and a lesser delay was observed for patients with ILD, 8.5 (2–84) months. Long-term enteropathy (n = 23, 9.6%) was noted as one of the infectious and non-infections complications that contributed to morbidity.

Management embraced frequent interaction with patients and their families. Extensive counseling was provided on nutrition, infection prevention, recognition of warning signs, adherence to action plans, and disease-specific treatment options. Proper assessment and follow-up of an individual clinical course for each patient were implemented. Most patients were evaluated by multidisciplinary teams for disease monitoring, complication prevention, and management. Management strategies included prophylactic antimicrobials (n = 142, 59.4%), intravenous immunoglobulin (IVIG) reconstitution therapy (n = 98, 41%), HSCT (n = 24, 10%), targeted immune therapy (n = 14, 5.9%), and biological therapy (n = 9, 3.8%). One patient with complete 22q11.2 deletion syndrome received thymic transplantation. At the time of the study, 92 patients (38.5%) were alive and well, and 66 patients (27.6%) were alive but with complications and morbidity. Seventy-six patients (31.8%) had been admitted to the ICU at least once, and 21 patients defaulted (8.8%). The management of immune dysregulation involved high-dose immunoglobulin therapy and topical and/or systemic immunosuppressive therapy with corticosteroids, interleukin-1, and interleukin-6 receptor antagonists, calcineurin inhibitors, mammalian Target of Rapamycin (mTOR) inhibitors, Janus kinase (JAK) inhibitors, and anti-CD20 monoclonal antibody. All patients with Lipopolysaccharide-Responsive Beige-Like Anchor Protein (LRBA) deficiency received abatacept.

The mean and median age of transplant was 32 and 19 months, respectively (range: 5–144). Among the 24 patients who received HSCT, 14 (58.3%) were reported to be in remission, 3 (12.5%) demonstrated rejection, and 2 (8.3%) had a relapse of original disease. Five patients are currently undergoing HSCT, with no conclusion of outcome at the time of this report. The donor source was a matched sibling in 12 patients (50%), haploidentical in 8 patients (33.3%), and a matched family donor in 4 patients (16.7%). More than half of the patients who received HSCT (n = 14/24, 58.3%) were transplanted in centers abroad, while 10 patients (41.7%) received HSCT in Oman.

**Figure 2:**
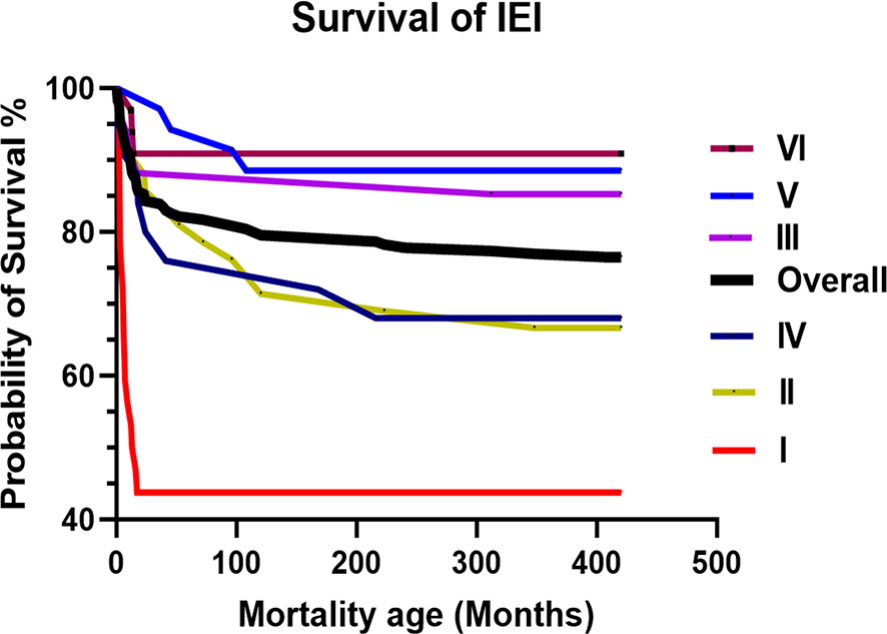
The overall and IUIS class-specific survival curve for Omani patients with IEI. Immunodeficiencies affecting cellular and humoral immunity (I). Combined immunodeficiencies (II). Predominantly antibody deficiencies (III). Diseases of immune dysregulation (IV). Congenital functional defects of phagocyte (V). Defects in intrinsic and innate immunity (VI). IEI, Inborn Errors of Immunity; IUIS, International Union of Immunological Societies.

The overall mortality rate was 25.1% (n = 60), with a mean and median age of 60 and 14 months, respectively (range: 1–408). The survival curve for patients with IEI is illustrated in **Figure 2**. Immunodeficiencies affecting cellular and humoral immunity (I) had the worst probability of survival at 44%, followed by syndromic CID (II) and immune dysregulation (IV) at approximately 68%. The causes of death included severe infection (n = 51, 21.3%), end-organ disease (n = 27, 11.3%), immunodysregulation complications (n = 13, 5.4%), do-not-resuscitate (DNR) order (n = 12, 5%), HSCT-related (n = 6, 2.5%), malignancy (n = 3, 1.2%), and others (n = 2, 0.8%). The bulk of the mortality was reported in class I (n = 20, 58.8%), followed by class II (n = 15, 30%), and class IV (n = 10, 37%). The mortality in class I was due to severe infection in 95%, uncontrolled immune dysregulation in 30%, end-organ damage in 30%, HSCT-related in 15%, and DNR order in 15%. The mortality in class II was due to severe infection in 93%, end-organ damage in 53%, HSCT-related in 40%, uncontrolled immune dysregulation in 26.7%, and DNR order in 6.7%. The mortality in class IV was due to uncontrolled immune dysregulation in 100%, severe infection in 60%, end-organ damage in 50%, HSCT-related in 10%, and DNR order in 10%.

## Discussion

The IUIS PID classification committee classified IEI into 10 major groups based on clinical and laboratory findings along with molecular diagnosis. The current study is the first to characterize 239 pediatric and adult patients with IEI in Oman and provides up-to-date detailed clinical and genetic data. The present results support a higher prevalence and incidence of IEI in such highly consanguineous community and poor survival. Early prevention and early management through screening programs for the classical T- and B-cell deficiencies as well as incorporating immunodysregulation as a warning sign for IEI are considered to represent effective approaches to improve survival.

In this study, we analyzed the demographic, clinical, and molecular data and outcomes of 239 patients diagnosed with IEI at the Royal Hospital. In line with the previously reported high prevalence of consanguineous marriage rate in Oman (49%) (21), greater than two-thirds of the cohort had a history of consanguinity. Therefore, one would expect to see more autosomal recessive and almost equal female-to-male distribution. However, this cohort had slightly more boys to girls (male/female: 58.1% vs. 41.9%), which is similar to the ESID registry (56% vs. 44%) (15).This finding may be because males are more commonly affected with X-linked diseases in addition to the autosomal recessive disease that affect both sexes in a similar proportion.

According to a previous Omani study, Al-Tamemi et al. (8) described an IEI prevalence of 7/100,000, while the current cohort reports a prevalence of 8.7/100,000. The overall calculated combined prevalence of this cohort and the Omani cohort included in the recent MENA report in 2021 (15) was 15.5 per 100,000 Omani population (**Table 1**). This emphasizes the importance of establishing a detailed national IEI registry in Oman.

Similar to previously published literature, infection was found to be the predominant clinical presentation, followed by immune dysregulation and then malignancy. We also observed that symptomatic recurrent bacterial infections were the most common clinical presentation with the respiratory tract being the predominant site of infection. Failure to thrive, chronic lung diseases, infective lymphadenopathy, and enteropathy were major complications of IEI in our group. This is in agreement with previous studies conducted in Qatar, Kuwait, and Egypt (5, 6, 22).

Interestingly, we found that immune dysregulation was prominent and occurred in approximately one-third of the patients (32%), which was a higher rate than that reported by previous studies from France (26%), Kuwait (20%), and ESID registries (18%) (23, 24). During the last 25 years, immune dysregulation was frequently reported in patients who suffer from IEI, and this association prompted further studies that led to discovery of new monogenic disorders, improvement in the knowledge of the pathogenesis of autoimmunity, and introduction of targeted treatments. The largest percentage of patients who showed clinical signs of immune dysregulation was in the category of diseases of immune dysregulation (41%), followed by PAD (18%) (**Table 3**). As depicted by Costagliola et al. (25), failure to eliminate self-reactive B cells, T-cell dysfunction, and reduced number and function of regulatory T cells (Tregs) are the main contributing factors in the development of immune dysregulation in such patients. A previous literature review showed that, regardless of what aspect of PID has been studied, early diagnosis reduces healthcare consumption and leads to better health outcomes in nearly all cases (26). Unfortunately, 35% of those who manifested with immune dysregulation died at a mean age of 50.8 months compared to the overall mean age of 41.7 months for the entire cohort. However, a mean difference of 9 months of delay is not statistically significant, highlighting the possibility of other factors such as uncontrolled immune dysregulation, ongoing end-organ damage, and/or treatment-related immunosuppression and effect of toxicity.

Another relevant finding was that the prevalence of both CID and SCID exceeded the frequency of congenital functional phagocyte defects in this cohort. A similar pattern was observed by Barbouche et al. (27) in Egypt, but was in contrast to other studies from the ESID registry (13), Iran (28), Kuwait (5), Tunisia (27), and Turkey (29), where other types of IEI were dominant. One possibility of the higher prevalence of these groups of immunodeficiencies in the current study compared to previous reported findings in the country (8) is related to the direct access and referral of different healthcare facilities to the Royal Hospital, which is considered the largest tertiary hospital in Oman. However, late recognition and referral hamper the appropriate care needed and often increase morbidity and mortality at peripheral hospitals.

We also found that 77% (n = 184) of this cohort had an attempt made toward obtaining molecular confirmation of their underlying IEI, with 50% (n = 120/239) having their genetic defect identified. Compared to the global molecular diagnosis rate of 13.2% (3) this higher percentage could be attributed to the higher rate of consanguinity in our cohort. The method of detection varies, but almost 90% were identified using whole-exome sequencing/next-generation sequencing and Sanger sequencing/targeted mutation analysis. The establishment of a molecular diagnosis has enabled the support of the suspected diagnosis and its expected spectrum of different IEIs. Moreover, molecular characterization guides the treating physician to establish a proper targeted and personalized clinical management, minimizing complications and allowing the provision of evidence-based genetic counseling.

Long-term follow-up of patients revealed that IVIG replacement therapy was conducted in 41% of patients, which is concordant with other cohorts from Germany (47%) (30) and Kuwait (58%) (5). HSCT was achieved in 10% of patients with IEI, among whom more than 56% underwent remission. The mortality rate in our cohort was 25.1% (n = 60), with a mean age of 60 months (range: 1–408). This is comparable to other reports of 26% in Kuwait (5) and 18.7% in Iran (28). A higher mortality rate was seen in patients with SCID/CID due to the delayed diagnosis. Similarly, patients with immune dysregulation had a mortality rate that was comparable to CID probably due to delayed diagnosis. Overall, the mean delayed diagnosis for all patients was 41.7 months, while it was 50 months for those with immune dysregulation. The lack of awareness that such patients is among the spectrum of IEI that has also contributed to the delay in diagnosis. As most of patients with IEI (SCID, CID and patients with immune dysregulation) are relatively asymptomatic at birth, in the absence of a proactive screening program such as a newborn screen program for T- and B-cell deficiencies, the presence of disease can easily be missed until manifestation. Indeed, the newborn screen program has been shown to be sensitive in detection by the new finding that Saudi Arabia has a SCID incidence of 1/2,906 live births (31) compared to the previous retrospectively reported 5.39/100,000 (7). The lack of adequate HSCT units in Oman that can urgently accommodate a sufficient number of patients also contributed in management delay, as most patients were transplanted abroad (58.3%). To overcome the aforementioned obstacles, our prospective proposition is to implement a national newborn screening program, recommend expanding the HSCT service in the country, establish a national IEI registry, and increase the awareness toward the phenotype and genotype of IEI in Oman.

## Conclusion

This study is the largest in the country and addresses important factors that lead to poor outcome in patients with IEI. Our findings acknowledge the need to include immune dysregulation as one of the warning signs of primary immunodeficiency to avoid delayed diagnosis of patients with non-infectious manifestations. The higher mortality rate seen in patients with SCID/CID highlights the urgent need for proactive detection through the establishment of a national IEI newborn screening program. Finally, the development of a national IEI registry is a priority, as it will help to unify the national efforts and develop effective short- and long-term strategies. Addressing the above issues will pave the way for better disease ascertainment, phenotype characterization, early diagnosis, and early intervention, thereby improving the likelihood of positive health outcomes for Omani patients with IEI.

## Data Availability Statement

The original contributions presented in the study are included in the article/supplementary material. Further inquiries can be directed to the corresponding author.

## Author Contributions

TF: Conception of idea, research design, data collection, data management, analysis, report writing, and intellectual input. KA: Data collection and data management. JA: Critical reviewing and report writing. MK: Critical reviewing and report writing. MC: Critical reviewing. AH: Reviewing genotype. ZA: Data collection and data management. IN: Data collection and data management. NS: Conception of idea, research design, data collection, data management, analysis, report writing, and intellectual input. All authors contributed to the article and approved the submitted version.

## Conflict of Interest

The authors declare that the research was conducted in the absence of any commercial or financial relationships that could be construed as a potential conflict of interest.

## Publisher’s Note

All claims expressed in this article are solely those of the authors and do not necessarily represent those of their affiliated organizations, or those of the publisher, the editors and the reviewers. Any product that may be evaluated in this article, or claim that may be made by its manufacturer, is not guaranteed or endorsed by the publisher.
